# Fetal Cerebral Blood Flow (Dys)autoregulation

**DOI:** 10.3390/diagnostics15202592

**Published:** 2025-10-14

**Authors:** Cristiana Moreira, Luís Guedes-Martins

**Affiliations:** 1 Instituto de Ciências Biomédicas Abel Salazar, University of Porto, 4050-313 Porto, Portugal; 2 Departamento da Mulher e da Medicina Reprodutiva, Centro Materno Infantil do Norte, Unidade Local de Saúde de Santo António, Largo Prof. Abel Salazar, 4099-001 Porto, Portugal; 3 Unidade de Investigação e Formação, Centro Materno Infantil do Norte, 4099-001 Porto, Portugal; 4 Instituto de Investigação e Inovação em Saúde, Universidade do Porto, 4200-319 Porto, Portugal

**Keywords:** Doppler, fetal cerebral circulation, fetal hypoxia

## Abstract

**Background**: As an extremely sensitive organ, particularly during in utero development, the brain has intrinsic systems to reduce the risk of cerebral damage in cases of insult, such as energy deprivation, due to a mechanism of positive balance in cerebral oxygen–energy substrate demand and supply. This mechanism is called cerebral autoregulation and is present in both the fetal and adult brain. The inaccessibility of the fetal brain to currently available measurement techniques limits its knowledge. Physiological and pathological alterations of fetal cerebral blood flow (CBF) can be assessed during the latter half of pregnancy using sonographic Doppler studies. The limited studies on this subject suggest a potential role for Doppler assessment of the fetal internal carotid artery. **Objective**: This article reviews the concept of CBF autoregulation and the role of fetal Doppler studies in various brain vascular territories in clinical practice. **Methods**: A PubMed search was performed, and 156 English articles were used as references in this bibliographic review, published between January 1996 and December 2021. **Results**: The study of fetal CBF involves indirect observation; the fetal brain constantly changes its characteristics towards complete maturation, which will be fully accomplished only after birth, and the maternal environment influences this process. **Conclusions**: Doppler study of the internal cerebral artery might be useful in clinical practice. However, technical issues for its study are not established, there are no reference curves, and studies on its clinical value have limited applicability.

## 1. Introduction

The development of the human brain begins soon after conception, when a portion of the dorsal ectoderm differentiates into neural ectoderm, forming the neural plate. The neural plate will fold longitudinally, giving origin to the neural tube, the primordial central nervous system. The encephalic mass will develop from the anterior portion of the neural tube and the spinal cord from its posterior portion [1,2]. After the closure of the neural tube, approximately 16 days after conception, a primordial system of endothelium-lined vascular channels, known as blood islands, composed of splanchnopleuric mesodermal cells, can be observed on the yolk sac chorion, marking the initiation of a process called vasculogenesis [3]. By the end of the embryonic period, a complex network of leptomeningeal arteries has developed to cover both rudimentary cerebral hemispheres. Posterior development of the brain vessels is dependent on its structural development, such as functional development of cerebral vasoreactivity [4].

Arterial and venous vasculature development as well as the maturational process are well characterized and described in five distinct steps. The developmental characteristics of the carotid artery system and other brain arterial systems were established by Lemire et al. in 1975, and correlated with crown–rump length and gestational age [5].

The first period of vascular development occurs within the first 20 days after conception, when vessels cannot be distinguished as arteries or veins, as they have the same appearance of primordial endothelium-lined channels. In the second phase of development, at 30 days of gestation, vasculogenesis and the beginning of angiogenesis give rise to the differentiation of discrete blood vessels, identified as arteries, veins, and capillaries, through the identification of communication with the aortic system and drainage from the brain via primitive head veins. A third moment of development is characterized by the segregation of the blood vessels into distinct systems. At 40–45 days postconception, three different vascular networks can be identified: external, dural, and cerebral. The cerebral network develops into the developing brain tissue with neurogenesis and angiogenesis occurring microscopically at neurovascular units [6]. During the fourth stage, there is a readjustment of blood vessels to the structural brain changes, which continues into the fetal period, showing the first similarities with the adult brain’s vascular vessels. The last period involves changes at the histological level, converting the walls of the vessels into their final adult form, a process initiated at 24 weeks of gestation and that continues 1 month of postnatal age in term newborns [6].

The development of cerebral arteries is intimately parallel to the development of the aortic arch system and is influenced by neural tube differentiation. In fact, after the development of the first and second arches, the primitive internal carotid and trigeminal arteries are found as branches of the first aortic arch. When those two first aortic arches involute, the primitive internal carotid arteries (ICAs) are extensions of the third aortic arch. Each internal carotid artery (ICA) will then branch into other smaller cerebral and extracerebral arteries, such as the dorsal ophthalmic and the primitive maxillary arteries. Further differentiation of the internal carotid system also gives rise to the middle cerebral artery (MCA). In parallel to the development of the early internal carotid system, other brain vascular systems develop, such as the external carotid artery system and the common carotid artery (corresponding to the stem of the internal/external carotid arteries), and the upper part of the basilar-vertebral system, which will later give rise to the posterior cerebral artery and the vertebral artery. In light of this knowledge, the abnormal development of segments of the cerebral circulation, specifically its vasoreactivity properties, may result from anomalous aortic arch development due to specific genetic or epigenetic signaling defects in neural crest development [5,7,8,9,10]. Neural crest cells are multipotent cells that contribute to the autonomic innervation of both systemic and cerebral vasculature, thereby affecting vasoreactivity during health and disease [11]. The embryological development of the common carotid artery explained before applies to the left side of the embryo, but not entirely to the right side, in which the right common carotid artery, together with the right subclavian artery, arises from the brachiocephalic artery and not directly from an aortic arch.

The vascular circle of Willis is completed with the development of the anterior and posterior communicating arteries. The complex pathway of development of the circle of Willis creates conditions for significant anatomical and histological variations, which might be reflected in responses to hemodynamic stress before and after birth [12]. In fact, preterm neonates show a high prevalence of variant types of the circle of Willis at term-equivalent age [13].

In parallel with the anatomical organization of the vascular brain network, a maturational process of angiogenesis occurs at the microscopic level within these primordial brain vessels, leading to the formation of the blood–brain barrier. Molecular and cellular mechanisms, defined by genetic factors, underlie the development of the neurovascular unit [5]. These genetically controlled mechanisms respond to environmental influences during prenatal maturation, resulting in either adaptive or maladaptive consequences [14].

Similarly, from the functional point of view, flow within the fetal brain circulation will also be influenced by systemic circulation, which, in turn, will be affected by environmental in utero circumstances, such as maternal, placental, and fetal health or disease [15]. This close functional connection is reflected in changes in placental vascular resistance, cardiac contractility, vessel compliance, and blood viscosity, all of which influence the dynamics of fetal cerebral circulation at different gestational ages in the fetus and neonate [4,16]. The fetal cerebrovascular system will then respond to local brain demands as well as to other extracranial modifications of fetal circulation, compensating for vascular and metabolic stresses by redistributing blood in situations of increased demand or a shortage of fuel resources [4].

Physiological alterations of blood flow in the brain and other organs can be assessed throughout the pregnancy using sonographic Doppler studies. Changes in pulsatility and resistance indices in fetal cerebral arterial vascular structures can be observed secondary to various physiological and non-physiological states of pregnancy, including fetal behavioral state transitions, alterations in breathing and heart rate, plasma glucose concentrations, and fetal head compression. For example, during fetal movements, namely, accentuated diaphragmatic movements, resistance in MCA decreases because an increased heart rate will cause dilation of cerebral vessels. Reverse flow patterns are part of a spectrum of abnormal diastolic flow patterns and occur at extreme conditions that adversely affect cerebral blood flow (CBF) integrity within the fetal brain [5].

The role of sonographic Doppler studies of MCA in assessing fetal well-being and response to prenatal and labor stress is well established [17,18,19,20,21,22,23]. Additionally, it is accepted that, in such circumstances, the MCA Doppler profile is one of the last to be affected, which is related to the conservation of essential organs, such as the brain, in extreme conditions [24,25,26].

Few studies in animal models or in preterm and term neonates show a hypothetical role for Doppler assessment of the fetal ICA, a more distal artery in relation to fetal cerebral circulation, similar to that offered by MCA [6,27,28,29,30,31,32]. We review in this article its development during the embryonic and fetal periods, its role in fetal cerebral circulation and blood flow redistribution, and ultimately, how its responses in pathological fetal or pregnancy conditions might be detectable in Doppler studies in fetal models.

## 2. Materials and Methods

PubMed was searched using the mesh terms “fetal cerebral”, “circulation”, “autoregulation”, “hypoxia”, “vascular contraction”, “fetal carotid arteries”, “signal transduction”, “blood flow”, “blood flow velocities”, “ultrasound”, and “fetal cerebral blood flow redistribution”, considering the subject of each topic of study. Publications were assessed for inclusion by one author (C.M.) following predetermined criteria. Inclusion criteria included publications (articles, books, and guidelines) written in English, published from January 1996 to December 2021. Articles published as abstracts were included if information was adequate to assess inclusion criteria and data on relevant outcomes were reported. Opinion articles were excluded.

**Figure 1:**
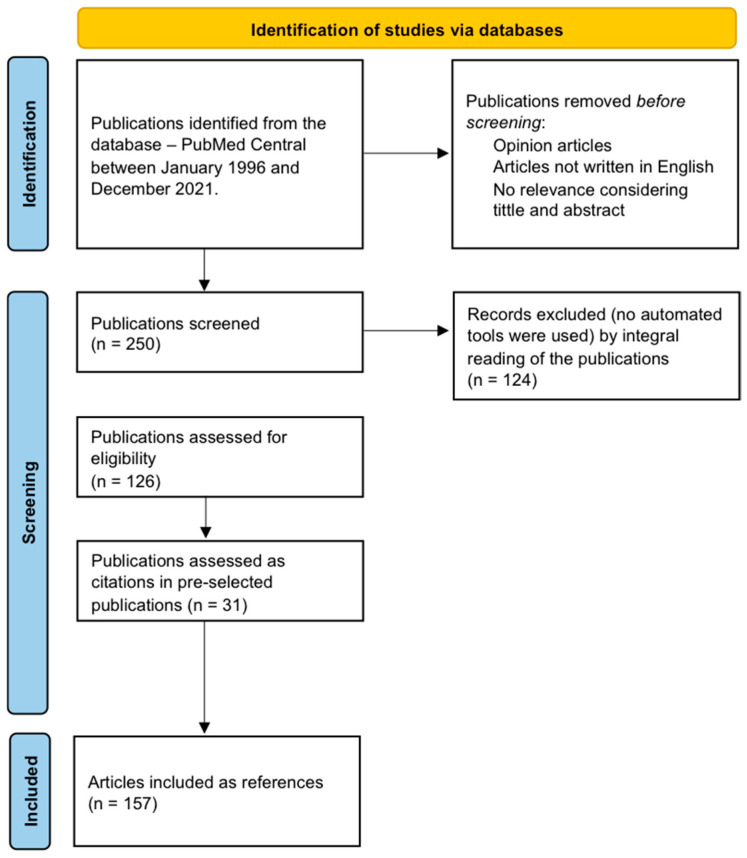
Methodology of article research and selection.

Two hundred and fifty articles were considered after reviewing the titles and abstracts of the articles, taking into account the relevance of the information for the intended review. Analysis of the selected sources implied an integral reading of the publications, and the authors extracted information on their results, discussion, and conclusion. Thirty one articles were added as references suggested in the articles initially read. Ninety-four articles were excluded after their integral lecture, as they were deemed not relevant to the subject. In the end, 157 articles were used as references in this bibliographic review, and the review focuses on the state of the art about fetal brain autoregulation, highlighting the lacunae in the field (Figure 1). 

## 3. The Fetal Cerebral Circulation

### 3.1. Fetal Cerebral Autoregulation

**Figure 2:**
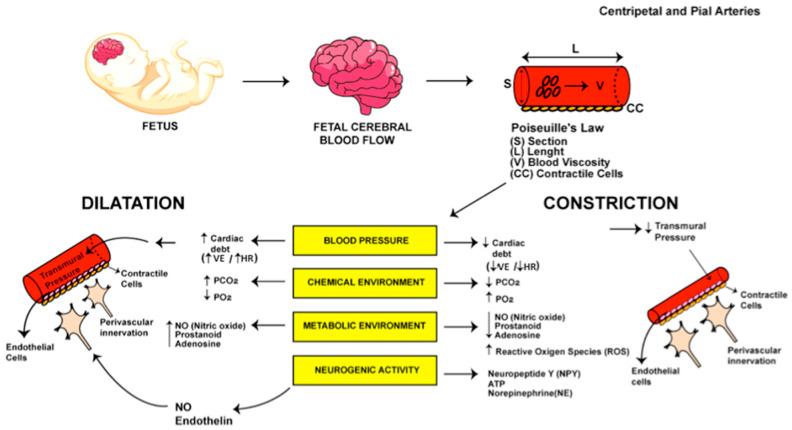
Autoregulation of cerebral blood flow: changes in section size and pressure gradient through the vessel, which is achievable by contractile and relaxation properties of those vessels and a function of blood pressure, chemical environment, metabolic environment, and neurogenic activity. ATP: adenosine triphosphate; CCs: contractile cells; EV: ejection volume; HR: heart rate; L: length; NE: norepinephrine; NO: nitric oxide; NPY: neuropeptide Y; pCO2: carbon dioxide arterial partial pressure; pO2: oxygen arterial partial pressure; ROS: reactive oxygen species; S: section; V: blood viscosity; ↑: increase; ↓: decrease.

As an extremely sensitive organ, particularly during in utero development, the brain has intrinsic systems to reduce the risk of cerebral damage in cases of insult, such as energy deprivation, due to a mechanism of positive balance in cerebral oxygen–energy substrate demand and supply. This mechanism is called cerebral autoregulation and is present in both the fetal and adult brain. The process of autoregulation of blood flow is based on the principles of Poiseuille’s Equation, according to which blood flow is dependent on four variables: the pressure gradient between the vessel, its section size, length, and blood viscosity. For a given blood viscosity and arterial length, the process of autoregulation depends on changes in vessel section size and pressure gradient through the vessel. It is achieved through the contractile and relaxation properties of these vessels [33]. In fact, arteries constrict in response to an increase in transmural pressure and dilate in response to a decrease in pressure, thereby maintaining stable blood flow within a range of arterial blood pressures. For this reason, this mechanism is also referred to as pressure-flow reactivity [16,34]. As shown in Figure 2, CBF depends on resistance of blood vessels and the perfusion pressure, which is created particularly by cardiac output in fetuses, but also by heart rate. Resistance of blood vessels is a result of vessels’ limited section size and blood viscosity, and is promptly modifiable by the variation in the diameter of arteries and arterioles in each organ through variations in blood pressure, chemical environment (carbon dioxide arterial partial pressure (pCO2) and oxygen arterial partial pressure (pO2)), metabolic environment (functional activation), and neurogenic activity [34]. In the cerebral territory, vascular resistance is primarily determined by arterioles, with contributions from the centripetal arteries that penetrate the cerebral mantle, as well as the pial arteries. It is assumed that shear stress at the endothelial level in the resting state is constant across vessel size and development. For this reason, the conduit arteries of the newborn brain will make a more significant contribution to vascular resistance than the germinal matrix pre-capillary arteries, since the muscular characteristics of pre-capillary arteries are not fully developed until late in fetal life [34]. The main differences between fetal and newborn CBF and autoregulation are evident at the moment of transition to extrauterine life. They are clearly influenced by gestational age at delivery and concomitant obstetric pathological conditions, reflecting the varying degrees of maturation of the fetal cerebral vasculature [35,36]. In fact, those differences in prenatal and neonatal autoregulation can also be explained in light of Poiseuille’s equation, assuming that, after transition, there are alterations in arterial pressure, blood viscosity (explained by hemoglobin concentration), and ultimately in the size of vessels, changed by a constantly developing and growing human being [33]. The fetal–neonatal–cardiopulmonary transition leads to a decrease in pulmonary vascular resistance associated with breathing movements, and an increase in fetal peripheral resistance [33]. This hemodynamic changes cause the closure of fetal shunts in the newborn [33].

The inaccessibility of the fetal brain to currently available measurement techniques limits our knowledge of fetal cerebral metabolic, neuronal, and vascular function. The current understanding is based on animal studies and studies in premature newborns, and has been developed since the 1970s [37,38,39,40].

The first animal model to study cerebral vasculature and blood flow was developed by Robert and Susan Vannucci [41] and has been used in several publications until recently [39]. Fetal lamb models showed a connection between fetal asphyxia and vasogenic brain edema [42], the reduced efficiency of cerebral autoregulation in the fetus when compared to the adult [16,43,44], and its vulnerability to hypoxic insults [39].

Similarly, studies on newborns of different gestational ages, healthy or with several pathologies, have been used to try to understand fetal brain behavior before delivery and its principal impact factors, namely, the consequences in newborns’ CBF during umbilical cord milking and clamp [45,46], fetal growth restriction [47], congenital heart diseases [47,48], the effect of maternal exposure to drugs [47], and chorioamnionitis [49]. Despite postconceptional age equivalency, there are differences in the internal and external environment of the fetus and premature newborn that limit the extrapolation of data, and studies on cerebral hemodynamics accomplished by near-infrared spectroscopy and, more recently, by diffuse correlation spectroscopy and arterial spin labeling have shown differences in fetal and neonatal responses to insult [36,50,51,52,53,54,55].

Although there are limitations concerning studies in animals and premature newborns, it is well established that restriction in fetal oxygen supply triggers endogenous fetal compensatory mechanisms at both systemic and cerebral levels, maintaining a positive energy balance. This occurs by decreasing energy demand and by increasing substrate supply. However, these adaptive responses are effective in a single dose of insult, losing efficiency when the insult duration or severity increases, and have a lower level of vulnerability in the fetus than in adults [4,39].

These adaptive mechanisms have an impact on fetal behavior and can be detected by fetal ultrasound (US), specifically through variations in the pulsatility index (PI) in cerebral arteries, as well as in the fetal electroencephalogram (EEG) [21]. In the central nervous system, the decrease in neuronal activity manifests as a decrease in fetal movements. This decrease in movement is accompanied by changes in the fetal EEG that are less energy demanding [4].

At the level of metabolic activity, a breakdown in oxygen or nutrient supply will impair the resynthesis of adenosine triphosphate (ATP), the energy source for metabolic well-being [4]. Adenosine, a breakdown product of ATP, accumulates, suppresses neuronal activation, and increases perfusion through vasodilatory effects, thereby enhancing fetal oxygen extraction. This mechanism is capable of sustaining fetal oxygen delivery until the umbilical venous oxygen content falls to around 50% of normal [4]. These responses differ to some extent depending on the mechanism of fetal hypoxemia and level of fetal maturation: if hypoxemia develops very slowly, fetal behavior may not decrease until acidosis develops [4,56]. Severe cerebral hypoxia will limit cerebral oxygen metabolism directly, leading ultimately to irreversible brain injury [4].

In hypoxemia, the net increase in CBF also triggers a redistribution of cerebral perfusion that favors the most metabolically demanding regions of fetal brain at a particular gestational age: brainstem perfusion exceeds cerebellar perfusion, which, in turn, exceeds blood flow to the cerebrum [4]. This robust vasodilatory response in the fetal brainstem makes it significantly more resistant to hypoxic injury than other brain regions [4,57]. This knowledge was the result of the application of a microsphere technique in the lamb model by the Ashwal and Longo team, who measured cerebral perfusion in different brain regions [4,57].

Facing the limitations of the pial window technique in neonatal piglets, which failed to show the independent responses of pial arteries facing different environmental insults [58], interest in isolated arteries increased. Ashwal and Pearce demonstrated the direct vasodilator effects of acute hypoxia on fetal cerebral arteries in vitro that varied with artery size and age [59]. Since the 1980s, Pearce W. and his work group in the Center of Perinatal Biology (Loma Linda University, Loma Linda, CA, USA) have been developing research in this field, and recently focused on the role of epigenetics in fetal vascular adaptation, not just in cases of chronic hypoxia, but also in various modifications of the in utero environment, such as maternal drug abuse and maternal comorbidities [39]. In fact, it is currently assumed, through the fetal programming hypothesis, that the in utero environment may influence the later development of adult disease. Similarly, during cerebral vascular brain development, genetically controlled mechanisms of maturation respond to environmental influences at the molecular and cellular levels, influencing angiogenesis and blood–brain barrier formation, resulting in adaptive or maladaptive consequences [60]. Together, these studies have proven helpful in understanding fetal brain function; however, the primary compensatory mechanisms of the fetal brain in vivo remain to be elucidated [4].

### 3.2. The Effects of Hypoxia on Fetal Cerebral Structure and Function

The adult brain comprises approximately 2% of total body mass [61,62,63,64]. Yet, it receives approximately 20% of cardiac output, which highlights the importance of an adequate nutrient and oxygen supply for normal brain function [61,62,63,64]. Moreover, the developing fetal brain may consume about 50% of the nutrient and oxygen supply, exceeding the demands of adult life [61,62,63,64].

Although the immature brain is capable of utilizing alternative energy substrates, which, in animal models, may support up to 60% of cerebral energy demand, the principal energy source for fetal cerebral development and function is glucose, primarily used in aerobic metabolic pathways with minimal production of lactic acid [64]. For aerobic metabolism to occur, besides glucose, oxygen delivery should be assured.

The energy supply of the developing brain must meet the demands required for its structural growth and maintenance, as well as those needed for functional neuro-axonal and glial activation [4,65]. The rapid development of synaptic, dendritic, and axonal elements in the cortical and subcortical gray matter leads to a significant increase in brain mass, a process in which cerebral oxygen substrate demands increase exponentially, particularly in the later stages of pregnancy [66]. These demands are met despite the low arterial pO2 of prenatal life, which is determined by the circulating oxygen content and blood flow, and is influenced by the integrity of the placental circulation [4,67,68]. In fact, cerebral energy requirements are initially low in early fetal life, but they escalate rapidly during the third trimester to support the function of enzymes, such as sodium–potassium adenosine triphosphate desidrogenase (Na/K-ATPase), which are critical for maintaining electrocortical activity and the propagation of action potentials [4,64]. In a term fetus with a body weight of 3.5 kg, brain weight of 455 g, and blood flow of 120 mL/min/100 g, CBF would be 546 mL/min or 35 percent of cardiac output [63,69,70]. The features of this period of accelerated brain growth have been described by quantitative in vivo magnetic resonance imaging studies in premature infants and, more recently, also in prenatal life [4,71,72,73].

In situations where oxygen availability is decreased, blood flow is redirected to favor vital organs, such as the brain and the heart, by increasing peripheral vascular resistance while decreasing cerebral vascular resistance [66,67]. This adaptive mechanism, known as “blood flow redistribution”, leads to both acute and chronic functional and structural adaptations and exhibits a limited response [4,66,68]. While initially CBF is maintained at the expense of other organs through local mechanisms of autoregulation and adaptive metabolic responses, in extreme oxygen deprivation, peripheral vasodilatation eventually occurs, triggering a hypotensive status and consequently hypoxic–ischemic cerebral damage [66,67]. This is usually an irreversible damage with lifelong morbidity, directly proportional to the duration, severity of oxygen deprivation, and gestational age at the time of the insult [66,67].

The relationship between hypoxia and the developmental stage has consequences on a cellular level. Recent studies suggest that hypoxia has different effects on the oligodendrocyte lineage at varying stages of development. Akundi et al. have suggested that very early oligodendrocyte precursors exposed to hypoxia undergo accelerated maturation, whereas hypoxia later in oligodendrocyte development triggers either degeneration or maturational arrest [4,65,74].

Hypoxia-inducible factor (HIF) is known to be the most important regulator of oxygen homeostasis, in both physiological and pathological conditions, as well as in both prenatal and postnatal periods of brain development [75]. HIF and its downstream target genes play a role in maturational processes, modulating cell differentiation, vascular development, angiogenesis, and metabolic homeostasis within the fetus and placenta [75]. Under mild hypoxic conditions, the HIF system is activated, modulating both immediate and delayed stress responses [75]. This pathway leads to activation of endogenous neuroprotective systems involved in apoptosis and anti-apoptosis, erythropoiesis, and angiogenesis. However, severe hypoxic stress alternatively triggers destructive processes through apoptosis and necrosis [75]. Consequently, acute and chronic responses of the fetal brain and circulation have different impacts on its development.

Several facts support the hypothesis that the umbilical venous oxygen supply is sufficient to support aerobic metabolism in fetal tissues even in early stages of hypoxia. In fact, during fetal hypoxemia, the umbilical venous–arterial oxygen extraction fraction increases from approximately 40% to 50–60% [76]. Conversely, under normal baseline conditions, supplemental maternal oxygen with fetal hyperoxia does not increase fetal oxygen extraction [77,78,79]. Finally, under normal circumstances, the umbilical venous–arterial lactate is not increased, and umbilical venous lactate may be higher, suggesting fetal lactate uptake [4]. Despite these facts, this equilibrium may be affected by disturbances in maternal–fetal circulation and fetal cerebrovascular function, with consequences for neurodevelopment due to the restriction of nutrient and oxygen availability. Acute or chronic abnormalities in CBF are considered a significant contributor to the pathogenesis of periventricular hypoxic white matter lesions in preterm infants [28]. These lesions are often associated with cerebral palsy and an adverse neurodevelopmental outcome [28].

There is a strong influence of oxygen delivery on oxygen consumption, and this coupling is reflected in the production of metabolic mediators that play an active role in the contractility of cerebral arteries, thereby contributing to changes in brain vascular response to acute hypoxia. In fact, apart from an increase in blood flow achieved through autoregulation to maintain oxygen delivery, there is an inhibition of oxidative metabolism and ATP synthesis, which affects protein synthesis and ultimately impacts synaptic plasticity and the generation of action potentials [80,81,82]. There is a well-established role for adenosine, prostaglandin (PG) E2, nitric oxide (NO), cyclic guanosine monophosphate (cGMP), and specific neuropeptides, such as serotonin (5-HT), in the vasodilation of cerebral arterioles during acute hypoxia [64,75,83]. Additionally, in these stressful circumstances, elevated fetal cortisol and catecholamines may be responsible for the shift towards an anaerobic state in the fetus [82,84], which affects cardiovascular and brain development and function due to their interference with tissue maturation and cell division [85,86]. The acute increase in peripheral vascular resistance as part of the blood flow redistribution mechanism is possibly due to an increase in endothelin-1-mediated vasoconstriction [87]; similarly, the acute decrease in cerebral vascular resistance might also be due to the direct effect of hypoxia in smooth muscle cells of cerebral arterioles and arteries by altering the influx of calcium (Ca^2+^) to its contractile cells, and by lowering the density and sensitivity of contractile agonist receptors [67]. Also, some human studies suggest that fetal cerebrocardiovascular adaptation to maternal stress, which is analogous to the stress of fetal oxygen deprivation, includes a decrease in cerebrovascular resistance [88]. Considering the fetal endogenous production of catecholamines during those circumstances in sheep [84], the reduction in cerebrovascular resistance may be associated with a lower density of alpha-adrenergic receptors in the cerebral circulation. Acidosis may also contribute to the decrease in cerebrovascular resistance [89]. On the other hand, maternal enhancement of perfusion achieved through physical exercise during pregnancy has a positive impact on the growth and function of the nervous system by stimulating cerebral aerobic mitochondrial metabolism [90].

Although the stress-induced vascular compensation mechanisms appear to spare the brain in sheep, fetal lacto-acidosis itself may directly affect neurodevelopment on a non-vascular level, leading to poor cognitive and neurodevelopmental outcomes. This raises the discussion about the effect of external corticoid exposure widely used for prenatal lung maturation in clinical practice. Although controversial, some studies show a transient cerebral hypoperfusion status after external corticoid in fetal sheep, causing diminished fetal movements and variability in fetal heart rate, which can also be seen in the human fetus. Those studies also show that, during that transient cerebral hypoperfusion, there is a loss of proteins involved in cerebral development and synaptogenesis [91,92,93], with potential long-term effects on brain development and an impact on programming of mental and behavioral disorders [80].

Chronic hypoxia affects systemic organ perfusion and function, as well as the cerebral parenchyma, vasculature, and neurovascular unit, which are consequences of long-term structural and functional changes resulting from alterations in gene transcription and protein expression [94]. At the systemic level, with sustained hypoxia, plasma hemoglobin concentrations rise and peripheral vascular resistance is attenuated, re-establishing blood flow to most fetal organs [67]. Both vascular endothelial growth factor (VEGF) and erythropoietin (EPO) are essential components of the endocrine response to chronic hypoxia, and, in turn, these factors bring about multiple significant changes in cardiovascular and cerebrovascular regulation [94]. Although fetal cardiac output is depressed by moderate chronic hypoxia, CBF is maintained by a sustained vasodilatory response [67,94]. Cerebral vasodilation contributes to lower carbon dioxide (CO_2_) blood levels and ultimately depresses ventilation [67]. Hypotension is also a consequence of acute hypoxia, and carotid chemoreceptors might play a role in this response [67]. The mechanisms for compensating for prolonged hypoxia are dependent on the integrity of maternal circulation [67]. For these reasons, placental insufficiency and maternal hypertensive diseases can lead to chronic deprivation of oxygen and nutrients, such as that observed in fetal growth restriction, where there is a severe, chronic decrease in oxygen and nutrient supply [67,72,95]. In this situation, the compensatory redistribution of blood flow may not be sufficient to maintain normal fetal brain development over prolonged periods [96,97].

From the upregulation of pontine adenosine receptors to the hypothalamic production of vasopressin and oxytocin, and release of atrial and brain natriuretic peptides, there are several sustained responses in fetal brain parenchyma to deal with prolonged hypoxia [64,81]. Directly at the vascular level, there is a high plasticity of developing arteries, which show heterogeneous sensitivity and responses reflecting differences in oxygen demands of different fetal brain regions [67]. Chronic hypoxia increases protein content in fetal cerebral arteries, depresses the magnitude of depolarization-induced contractions, and also depresses the densities of several receptor types that drive contraction in these arteries [64,81]. In this context, the hypoxic fetus is more delicately balanced between contraction and relaxation than in situations of normal oxygen delivery. These adaptations to chronic hypoxia aim to conserve energy while preserving basic contractility [64,81]. A necessary consequence of these vascular effects is that the smaller and more peripheral cerebral arteries relax wholly and quickly in response to hypoxia [67]. In contrast, the larger and more proximal arteries, including the common carotid, maintain tone more effectively and play a more significant role in the gradual adjustments of cerebrovascular resistance to hypoxia [67]. Indeed, it is known that the brainstem is the most sensitive fetal brain region to oxygen deprivation and, consequently, is responsible for initiating vasodilatory responses and neovascularization in that situation [67]. However, it is also the most resistant region of the fetal brain when facing hypoxia [67].

Perivascular nerves, forming neurovascular units, exert motor, sensory, and trophic influences on the smooth muscle and endothelial constituents of the arterial wall [67]. Consequently, chronic hypoxia also has an effect at this level [67]. For example, chronic hypoxia depresses the function of NO-releasing nerves in the MCA, due to decreased expression of neuronal nitric oxide synthase (NOS). Because NO can facilitate norepinephrine (NE) release from adrenergic nerves, overall NE release decreases in chronically hypoxic fetal cerebral arteries. This response to hypoxia is absent in adult cerebral arteries [67,81]. Some studies on the neonate brain hypothesize that the permeability of the blood–brain barrier might also be affected by oxygen deprivation, through the depression of endothelial NOS messenger ribonucleic acid (mRNA) and protein levels [67].

Without doubt, chronic hypoxia brings about a diverse sequence of adjustments in neuronal and glial protein expression and regulation within the fetal brain. How these changes are coordinated and how they influence overall cerebrovascular regulation remain largely unknown.

### 3.3. Contractile Characteristics of Fetal Cerebrovasculature

Autoregulation in cerebral arteries of both fetus and adult, namely, under hypoxic conditions, is a consequence of the intrinsic characteristics of these particular vessels and the influence of external and environmental influences. The primary purpose of autoregulation is to establish equilibrium between CBF and the metabolic demands of brain tissue. CBF is influenced by transmural pressure gradients, shear stress, and perivascular neuronal activity through chemical, endocrine, and metabolic factors, originating in the brain parenchyma and blood, and representing the effectors of the cerebral autoregulation cascade [98].

Those stimuli are expressed differently in fetal and adult life. Additionally, the autoregulation capacity is influenced by the maturational status of cerebral vessels. Consequently, in fetal life, immature arteries and autoregulation responses make the fetal brain more prone to injury, such as intracerebral hemorrhage, with an increased risk inversely proportional to the degree of prematurity [98].

The recognition of cerebrovascular involvement in neonatal brain injury stimulated studies on cerebrovascular maturation, both structurally and functionally, the role of the neurovascular unit, and the influence of maturation on the regulation of CBF [98].

Most of the brain tissue, specifically the telencephalon, lacks vascular structures during early pregnancy. By the seventh week of gestation, endothelial channels with simple walls of endothelial cells originating on the primitive leptomeningeal plexus penetrate the brain tissue [99]. Muscularization will occur in striatal arteries early in gestation [99]. At the same time, most extrastriatal channels will develop into large sinusoidal channels, maintaining their endothelial characteristics, without apparent muscularis until the final weeks of gestation [99].

Myogenic vascular reactivity is attributed to the activity of cells with motile properties, specifically the vascular smooth muscle cell layers [3]. In the developing brain, smooth muscle cell layers form at approximately 20–22 gestational weeks around primordial vessels, which later give rise to the pial arteries, as well as around the superficial penetrating vessels [99,100]. The process of muscularization parallels the maturation of brain development, occurring in the rest of the cerebral arterial tree from these surface arteries into deeper parenchymal vessels, with some authors reporting that the process is not completed until after term birth [4,99]. That justifies why, in preterm newborns, the risk of encephalopathy and cerebral hemorrhage is higher, relating those outcomes to the limited control over the cerebral vasculature resistance [3].

Despite the role of the muscular layer in the resistance of cerebral arteries, endothelium-dependent regulation of arteriolar diameter and blood flow will remain an essential contributor to the autoregulation capacity of cerebral arteries [101]. Also, apart from the muscularization process, maturation brain arteries acquire contractile properties because smooth muscle cells also suffer a molecular maturation process, thereby developing a network of characteristic membrane receptors and channels that make them directly or indirectly (via complex signaling transducing mechanisms) responsive to the intrinsic and extrinsic characteristics described above: pressure gradients, shear stress, and perivascular neuronal activity as well as chemical, endocrine, and metabolic factors [102].

### 3.4. Fetal Cerebrovascular Signal Transduction

Interest in CBF and the autoregulation process arises from its known role in clinically relevant conditions, such as hypoxia and cerebral asphyxia related to pregnancy and delivery [98]. In fact, much of the knowledge about this topic comes from studies of hypoxic status in animal models [98].

Patterns of fetal and neonatal brain injury are known to be different from adult brain injury [98]. They are associated with high rates of morbidity and mortality, emphasizing the need to understand the biochemical and molecular basis of these differences [98]. Cerebrovascular involvement in neonatal brain injury and its particularities, namely, the role of cerebrovascular structural and functional maturation in regulating CBF in parallel with metabolic demands, and the role of the neurovascular unit, are subjects of interest in scientific research [98]. Signaling transduction mechanisms involved in cerebrovascular contractility and their changes with maturational development have been studied over the last few decades [98]. However, investigation into cerebrovascular maturation is complicated by the heterogeneity observed among vessels from different species, arteries of varying sizes, and among different vascular beds [98]. Additionally, nutritional and health status may have a significant impact on vascular characteristics and development [98]. The cerebral vascular phenotype is, in fact, highly dynamic, reflecting the changes in its environment during maturation processes [98,103].

**Figure 3:**
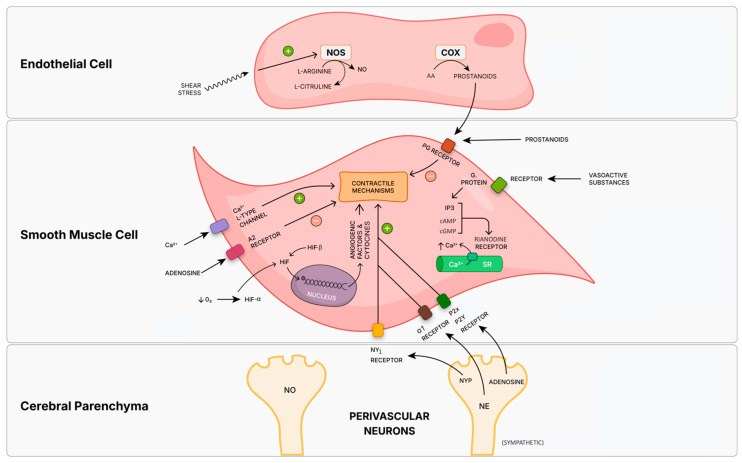
Signal transduction mechanisms of vascular contractile mechanisms. AA: arachinoid acid; Ca^2+^: calcium; cAMP: cyclic adenosine monophosphate; COX: cyclo-oxygenase; cGMP: cyclic guanosine monophosphate; HIF: hypoxia inducible factor; NO: nitric oxide; NOS: nitric oxide synthase; O_2_: oxygen; PG: prostaglandin; SR: sarcoplasmic reticulum; ↑: increase; ↓: decrease.

The differences in reactivity of the arteries in the fetal brain are a consequence of the presence of different cells in the artery wall with specific patterns of differentiation: vascular endothelium, smooth muscle cells, adventitia, sympathetic perivascular nerves, and parenchyma. As a consequence of the heterogeneous cell types constituting the artery wall, there is also a complex combination of signal transduction pathways in each cell type and artery: endothelium-mediated prostacyclin and eicosanoid; NO and cyclic nucleotide relaxation mechanisms; Ca^2+^-dependent contraction mechanisms, either through receptor–second messenger coupling with plasma membrane potassium (K^+^) channels and Ca^2+^ channels, or intracellular Ca^2+^ stores (Figure 3) [98].

Three basic developmental pathways can occur in elements of the fetal brain: elements might remain constant, gain a more prominent role over the course of maturation, or their role might be attenuated [98]. During normal development, vascular responses remain similar, with a slight change in contractility capacity but a larger magnitude of change in receptor density [98]. This promotes relaxation (the hyper-relaxation hypothesis) and attenuates contraction (the hypo-contractile hypothesis), the principle underlying the cerebral redistribution mechanism [98]. Because of the fetus’s limited ability to alter vascular contractile status, it is more vulnerable than the adult to alterations in oxygen availability and increases in hydrostatic pressure [98].

Vascular contraction, through contraction of smooth muscle cells in the arterial wall, is initiated by Ca^2+^ release from intracellular organelles, mediated by the synthesis and release of the second-messenger inositol-1,4,5-trisphosphate (IP3) [104,105]. Extracellular Ca^2+^ also plays a role in intracellular Ca^2+^ release via membrane ion channels and the activation of ryanodine-sensitive receptors on the sarcoplasmic reticulum (SR), a process called Ca^2+^-induced Ca^2+^ release [104]. The most robust intracellular Ca^2+^ source, both by ryanodine- and IP3-sensitive Ca^2+^ release, is the SR [104]. The relation between receptor activation and Ca^2+^ release is not constant, suggesting that physiological and pathological stimuli might influence the interaction between receptor or ion channel activation and Ca^2+^ release [104]. The role of intracellular Ca^2+^ stores and their sensitivity to various agents may differ significantly depending on vessel type, species, and developmental age [105].

#### 3.4.1. Calcium

One of the most critical differences in cerebrovascular contraction mechanisms between fetal and adult is, in fact, the apparent dependence of the immature fetal organism on extracellular Ca^2+^, showing greater Ca^2+^ sensitivity [98,106,107,108]. In contrast, adult contractile cells use mainly intracellular Ca^2+^ stores [98,106,107,108]. In the immature brain, L-type Ca^2+^ channels and K^+^ channels (in particular the BK channel) play an essential role in regulating Ca^2+^ entry into the cerebral smooth muscle cells [104,105]. Additionally, intracellular Ca^2+^ stores are limited in immature cells, demonstrating much less releasable Ca^2+^ than adults, highlighting the dependence of fetal cerebral arteries on extracellular Ca^2+^ [105].

However, the time during the course of development when the SR becomes a prominent Ca^2+^ store and the cerebral vessels become less dependent on extracellular [Ca^2+^], as well as the molecular mechanisms behind this transition, remain to be explained [98,105].

#### 3.4.2. Nitric Oxide and Prostaglandins

NOS is an enzyme present in vascular endothelial cells, participating in the regulation of CBF and neurotransmitter release, as well as serving as a second messenger in glutamatergic and cholinergic systems. NOS activity becomes more important with progressive neuronal maturation and metabolism development, increasing from mid- to late gestation, contributing to significant increases in CBF [63]. Geary et al. studied vascular diameter and intracellular concentration of Ca^2+^ as a function of intravascular pressure in preterm, near-term ovine fetuses, pressurized resistance-sized cerebral arteries (~150 μm), and non-pregnant adults. Cerebral vascular tone at term was dramatically altered after inhibition of NOS, cyclo-oxygenase (COX) with indomethacin, and endothelium removal, proving the critical role of endothelium-dependent mechanisms [101,109]. Experiments conducted in utero have also shown that raising intracranial pressure decreases cerebrovascular resistance in the near-term but not mid-gestation fetus; this is due to NOS and COX modulation mechanisms at term that provide a cerebrovascular reserve capacity, maintaining CBF during reductions in cerebral perfusion pressure through a vasodilatation effect [109]. In fact, findings from studies in different species indicate that NO becomes increasingly more important as a contributor to endothelium-dependent dilation in the pulmonary, cerebral, and skeletal muscle vascular beds [101,110]. Studies about hypoxia-mediated vasodilation of CBF also show a role of other molecules in the induction of ischemic tolerance, such as heat shock proteins, reactive oxygen species (ROS), nuclear factor-B, adenosine A1 and A2 receptors, and ATP-sensitive potassium channels [111,112].

#### 3.4.3. Adenosine

Adenosine A2A receptors are thought to be involved in hypoxia-induced angiogenesis via autocrine, paracrine, and hormonal vascular responses [113]. Adenosine has a direct vascular action, as well as indirect relaxant effects on A2A-mediated release of arginine vasopressin, which is also a vasodilator in fetal cerebral circulation [112,113].

#### 3.4.4. Reactive Oxygen Species

In hypoxic conditions, direct hypertension, shear stress, or the indirect action of angiotensin II (ANGII) leads to the overgeneration of ROS in the vascular wall [114,115,116,117]. ANGII is a vasoactive peptide produced by activation of the renin–angiotensin system and it is involved in the stimulation of NAD(P)H oxidase, and might also influence the production of mitochondrial ROS [114,115,116,117]. Stimulation of cerebral RAS activity triggers several downstream signaling pathways that may stimulate sympathetic nervous system activity, culminating in vasoconstriction not only in the cerebral territory but also in peripheral arteries, thereby justifying the development of hypertension later in infancy and adulthood [114,115,116,117].

#### 3.4.5. Hypoxic Inducible Factor-1

Under normal oxygen cell delivery conditions, hypoxic inducible factor 1 alpha (HIF-1α) is rapidly ubiquitinated and targeted for degradation in the proteasome [118]. During hypoxia, the oxygen-regulated HIF-1α subunit stabilizes, accumulates, and dimerizes with the constitutively expressed HIF-1β subunit [119]. This dimer eventually favors the transcription of multiple genes that encode angiogenic cytokines [120]. Coupling between HIF and angiogenic factors, such as EPO, VEGF, platelet-derived growth factor (PDGF), and fibroblast growth factor (FGF), as well as their respective receptors, maintains the supply of oxygen and nutrients to all cells [106]. These compensatory mechanisms increase vascular density to postpone an oxygen deficit and adapt the metabolism to hypoxia, potentially representing an interaction between neuronal and non-neuronal mechanisms of cerebral vasodilation [106]. For example, VEGF receptors are present in sympathetic nerves, and their activation induces proliferation and differentiation of neural cells, indirectly contributing to both structural and functional hypoxic remodeling of the fetal cerebral vessels [106].

#### 3.4.6. Neurovascular Unit and Its Effectors

While non-neuronal factors and their effects on vascular cerebral cells are the subject of a wide variety of studies in the literature, the trophic role of perivascular nerves is less well understood. Regulation of vascular tone in response to neuronal activity also plays a crucial role in controlling blood flow during development.

Through pathways such as those described above, hypoxia increases sympathetic innervation. Consequently, release of norepinephrine, neuropeptide-Y (NPY), and ATP is potentiated [106]. Those molecules will eventually influence smooth muscle phenotypes on cerebral arteries [106]. Neurovascular coupling describes the interaction between CBF, neuronal activity, and the metabolic demands of these activities. A focal increase in CBF occurs in response to local neuronal activation, reflecting the metabolic needs of neuronal status [106]. This cerebrovascular reactivity enables the brain to maintain homeostasis and physiologic function and to adapt to hypoxia, altering the function of contractile proteins and electrical behavior of the smooth muscle membrane [83].

NE is the primary neurotransmitter released from postganglionic sympathetic nerve terminals, interacting with vascular smooth muscle cells during acute hypoxia. This acute hypoxic upregulation of sympathetic nerve activity leads to local release and increased activity of NE [106]. In turn, Long et al. (2022) found that chronic hypoxia can attenuate lightly the ability of NE to induce vascular contraction through the downregulation of adrenergic receptor density [83,103]. This response is dependent on the maturation degree, being greater in adult (18%) than in fetal arteries (3%), because fetal arteries have great receptor reserve for both receptors of NE [83,103]. This also reflects the differences in quality and intensity of response in sympathetic cerebral nerves of adult and fetus, due to differences in density, neurotransmitter content, release and reuptake capacity, cleft width, and rates of neurotransmitter degradation [83,106,121]. Trophic actions of NE are highlighted when sympathectomy is performed, culminating in extracellular matrix remodeling and promoting the switch of smooth muscle contractile phenotype to a non-contractile one.

NPY is another potent vasoconstrictor released from sympathetic postganglionic nerve terminals after activation of Y1 receptors [67,83]. Apart from contractile activity, and similarly to NE, NPY has trophic and mitogenic effects on smooth muscle, being able to promote angiogenesis through activation of Y1, Y2, and Y5 receptors [67,83].

ATP is also released from sympathetic nerves and exerts its effect on cerebral arteries by activating the P2 class of purinergic receptors, which are coupled to either ion channels (P2X) or G-protein-coupled receptors (P2Y). Acutely, ATP mediates vascular contraction and contributes to mitogenic and trophic effects in the long term [112,113].

5-HT is also involved in neurovascular coupling in cerebral arteries. Teng et al. (1998) showed that the dominant 5-HT receptor in ovine cranial arteries is the 5-HT2a subtype [122]. The effect of hypoxia varies depending on the cerebral territory. In contrast to NE receptors, its expression remains stable under chronic hypoxia in middle cerebral ovine arteries [122]; however, under long-term oxygen deficit, the downstream signaling pathway initiated by the 5-HT2a receptor distinctly undergoes alterations from that observed for the α-adrenergic pathway [122]. The net effect of chronic hypoxia appears to be neutral in 5-HT-induced contractions of ovine MCA, both in the fetus and in the adult [122]. However, under the same circumstances, there is a decrease in 5HT2a receptor density by 73% and 49% in adult and fetal carotid arteries, respectively [67,102,103,122]. For this reason, the magnitude of the 5-HT-induced contractility was not decreased by chronic hypoxia in adult carotids, but was decreased by 52% in fetal arteries [67,102,103].

The regulation of CBF under hypoxic conditions, as described above, reveals the complex interaction between regional vasodilator and vasoconstrictor synthesis, neuronal and non-neuronal effectors, and intricate signaling pathways, highlighting its dynamic maturational characteristics.

### 3.5. Fetal Carotid Arteries Maturation

Normal brain development implies normal oxygen and nutrient delivery, which are dependent on the normal development and function of both the placental circulation and the fetal systemic and cerebral circulations [4]. The first vascular component of the embryo develops at 4 weeks of gestation, immediately after closure of the neural tube, in the form of a primordial system of vascular channels lined by endothelium [4]. During the transition to the fetal period, the primordial cerebral hemispheres are already irrigated by a large mesh of leptomeningeal arteries. Subsequent vascular development and reactivity are dependent on the structural and metabolic demands of the brain parenchyma [123]. However, the ICA develops differently [123]. In fact, it has a primordial origin and, together with the basilar artery, forms one of the two main arteries supplying blood to the brain [123]. Age-dependent properties of these arteries might explain the changes in distal vessels and in brain parenchyma during brain development in the fetus [123].

During delivery, there is a rapid change in the gas exchange platform, from the placenta to the lungs. With lung expansion, exposed to atmospheric pressure, there is a decrease in pulmonary vascular resistance and pressure, accompanied by a parallel increase in systemic vascular resistance and, consequently, in arterial blood pressure [123]. Initially, left cardiac output increases to offset the rise in peripheral resistance, but slowly decreases to reach a plateau. During these extensive alterations in cardiac function and vascular resistance, brain blood flow is maintained at an optimal level to ensure cerebral oxygenation and metabolism [123]. The role of carotid arteries in this process has already been proven, related to their contractile ability, which defends distal cerebral vessels from injury associated with high blood flow [123,124]. This process is not yet well developed in the preterm fetus, which can explain the increased vulnerability to cerebral hemorrhage, related to rupture of distal fragile vessels [123].

The transition from intrauterine to extrauterine life is also accompanied by dramatic changes in circulating vasoactive hormones and metabolites. The increase in NE and epinephrine, cortisol, prostaglandins F2α, I2, and D2, and ANGII, and decreases in circulating adenosine and PGE2, potentiate the effect of cerebral vascular contraction capacity and blood flow redistribution to rapidly face hemodynamic changes immediately after birth and protect the newborn brain from the increase in arterial pressure that happens in this transition [124,125]. This justifies why preterm newborns are more prone to brain hemorrhage.

We have already explored the maturational changes in the properties of vascular smooth muscle cells, namely, the dependence of fetal smooth muscle cells on extracellular Ca^2+^ for contraction mechanisms and the increasing importance of the SR as a source of Ca^2+^ as term approaches. As previously presented, adenosine plays a role in preserving cerebral oxygen and nutrient delivery by improving blood flow and suppressing metabolism through the A1 receptor. It was also demonstrated that adenosine has a similar effect, specifically on the carotid artery in fetal sheep, by an A1 receptor agonist, causing selective vasodilation in this vessel [126]. However, the global impact of this process is weak because the A1 receptor agonist reduces blood pressure, limiting blood flow to the cerebral territory [126].

From an enzymatic point of view, it is known that the activity of myosin light chain kinase (MLCK), a rate-limiting enzyme for vascular contraction, is similar in fetal and adult ovine carotid arteries. However, Sorensen et al. (2020) showed that, in relation to cellular compartmentalization of that enzyme, the adequate quantity of MLCK available for phosphorylation of the myosin light chain (MLC) 20, directly involved in contraction, is significantly greater in fetal arteries than in the adult ones [127]. Rejeti et al. also concluded the same for ovine carotid arteries [128]. Royal and De Longo (2011) demonstrated in studies of sheep carotid arteries that the fragility of parenchymal cerebral arteries in the premature fetus can be related to the low quantity of collagen in those vessels at that stage of development [129]. The same group also studied the influence of isoforms A and B of filamin (FLNA and FLNB) in brain development, showing that these actin cytoskeleton pathway gene expressions, particularly for FLNA, are downregulated in early fetal life and become progressively more expressed in the near term [129]. Another gene product evolved in the smooth muscle cell phenotype, switching to a contractile type, is formin binding protein 1 (FBP1), whose downregulation is attenuated during fetal development [129].

These events reflect the maturation process, aiming to prepare the fetus for the extrauterine environment [129]. The net effect of all those molecular maturational changes will be the transition to a contractile phenotype in smooth muscle cells of cerebral fetal arteries. It is expected that these changes will be mirrored in spectral Doppler analysis of the ICAs, similarly to what happens in the MCA. In MCA, in parallel with placental aging from early fetal life to term, resistance parameters, such as the PI, decrease, reflecting a decline in cerebral vascular resistance that maintains blood flow to this vital organ [129]. Contractile ability will be crucial in protecting cerebral distal and fragile blood vessels from the high-pressure blood flow that occurs immediately after birth. What exactly happens in the ICAs, which are more proximal to the heart and have a different embryologic origin, is not well characterized in the literature yet.

### 3.6. The Concept of Fetal Cerebral Blood Flow Redistribution

In parallel with the autoregulation process achieved in cerebral circulation, peripheral circulation also adapts to cerebral blood flow demands, enabling the autoregulation process to be entirely possible. The organs favored in this process are crucial to survival and include the brain, the heart, the adrenal glands, and the liver [4,130]. The optimization of their arterial blood flow is achieved by a parallel reduction in the arterial blood flow in other organs. This process is called blood flow redistribution.

Oxygen deprivation of various origins is the classical example of a blood flow redistribution trigger and one of the most common challenges in prenatal life. While short episodes of oxygen deprivation are easily tolerated through blood flow redistribution activation, for example, during labor and delivery, in chronic hypoxia, redistribution mechanisms may be ineffective, particularly in maternal, obstetric, and fetal conditions characterized by placental resistance [131]. While adults have a wide range of oxygen delivery through pulmonary function, in the fetus, oxygen delivery is restricted to the placenta and is dependent on its appropriate function, as well as on pre-placental ventilatory and cardiovascular maternal function, and on fetal health to ensure oxygen reaches vital organs [4,131]. For this reason, compensatory mechanisms in fetal life are well established to cope with the limitations in oxygen delivery, namely, the high capacity of fetal hemoglobin to bind oxygen; the capacity to deliver oxygen in tissues at lower oxygen levels; the presence of fetal shunts, such as ductus arteriosus, ductus venosus, and foramen ovale, which optimize the circuit of oxygenated blood; and redistribution mechanisms [131]. These adaptations are clinically illustrated by bradycardia and diminished fetal movements during periods of transient oxygen delivery failure, which demonstrate the optimization in oxygen consumption achieved through cardiovascular and metabolic adaptations [131]. In fact, the production of metabolites, such as lactate in response to hypoxia in tissues of non-vital organs, due to the blood flow redistribution mechanism, potentiates the release of oxygen in the tissues. Fetal heart rate decrease allows for an increase in ventricular filling and output to vital organs. However, the intrinsic cardiac capacity to increase ventricular output limits the heart’s response when hypoxia is not acute. In this circumstance, sympathetic tone is increased as a function of chemoreceptor activation, resulting in peripheral vasoconstriction in non-vital organs and an increase in umbilical oxygenated venous blood flow to the ductus venosus [4,69,131] initiating the redistribution mechanism. Cardiac output is then increased through augmentation in cardiac preload and a brain-sparing effect is possible to fulfill [4,21,131].

Autoregulation in CBF can be interpreted as a function of blood flow redistribution. The relationship between blood flow in the fetal brain and blood flow in other organs can be detected in prenatal US through the pattern of fetal growth and also by well-established spectral and color Doppler studies, with particular value in high-risk pregnancies. The redistribution of blood flow in high-risk pregnancies of various etiologies may be reflected in asymmetric growth restriction, which results from normal blood flow to the fetal brain at the expense of diminished blood flow to other territories, such as abdominal organs and limbs [21].

Despite the knowledge about compensation mechanisms to hypoxia and fetal brain-sparing effect in such conditions, studies also show its limited capacity in the prevention of brain damage and clinically relevant neurodevelopmental consequences [18,96,132,133,134,135], highlighting the importance of future studies in other brain vascular territories, besides the well-known umbilical and middle cerebral arteries [136,137,138].

### 3.7. Doppler Studies of Foetal Cerebral Blood Flow

Understanding fetal CBF is an essential step in gaining a better understanding of the pathogenesis of perinatal brain damage [6]. The recognition of the adaptive mechanisms triggered when deviation from fetal metabolic and hemodynamic equilibrium occurs has limited importance unless it is clinically applicable. The aim is to detect in utero pathological conditions and to improve medical management and intervention, minimizing in utero mortality and prenatal iatrogenic morbidity. In fact, the detection of the arteries that first show evidence of brain-sparing mechanisms early in hypoxia could help prevent the consequent neuronal damage [137].

While the majority of adult vascular territories are easily accessible to be studied, the study of fetal CBF in vivo, which is in utero, poses a challenge essentially for four reasons: (1) it is an indirect observation, (2) fetal brain is constantly changing its characteristics towards complete maturation, (3) maturation of fetal brain will be concluded only after birth, and (4) it will change according to the maternal environment [139].

**Figure 4:**
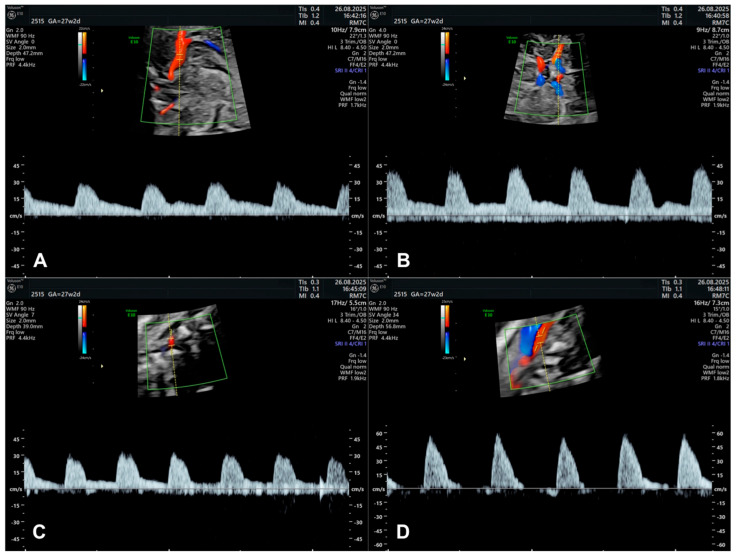
Normal velocity waveforms of different arterial territories at 27 weeks and 2 days of gestation (same patient), obtained by power Doppler evaluation recorded according to ISUOG Guidelines: Use of Doppler velocimetry in Obstetrics (2021). (**A**) **Anterior cerebral artery** (ACA): The waveform can be obtained in a sagittal plane and the vessel can be identified with color Doppler application, and should be obtained in the proximal segment of the artery. (**B**) **Middle cerebral artery** (MCA): The waveform can be obtained in an axial plane, in the proximal segment of the vessel. (**C**) **Vertebral artery**: Identified in a sagittal plane, running parallel to the spine. (**D**) **Left internal carotid artery** (ICA): Identified in paraxial plane, on each side of the neck, right below the jaw. Differences in maximum velocity in systole and diastole can be observed: internal carotid artery, more proximal to the heart, shows no diastolic flow and higher maximum velocity in systole; anterior and middle cerebral arteries show a positive diastolic flow with a lower maximum velocity in systole, findings associated with higher distance to the heart and intrinsic properties of these vessels. In pathologic conditions, autoregulation will accentuate those characteristics, reflecting the capability of decreasing resistance to blood flow.

Initial studies on fetal CBF were conducted in instrumented fetal sheep preparations and using adult functional imaging techniques [6,140]. In 1979, Bada et al. used Doppler ultrasound to assess the anterior cerebral artery (Figure 4A) through the anterior fontanel, and compared CBF velocity waveforms in both normal newborns and those with pathological conditions [6,141]. Nowadays, Doppler ultrasonography is a widespread technique for the assessment of placental and fetal cerebral circulation, and the literature shows a wide variety of clinical applicability [142]. Because the fetus lies in amniotic fluid, assessing CBF does not require a specific window, as it is easily accessible in neonatal life [6,143]. Some particularities should be taken into account when studying cerebral vasculature that physiologically change CBF waveforms on Doppler studies or can be a reflection of pathology. In fact, anatomic variations and anomalies in the arterial components of the circle of Willis have been detected in adults with cerebral aneurysms, hemorrhagic and ischemic lesions, as well as in normal individuals. In the fetus, the role of these variations is to be determined, but they might alter normal Doppler indices [6,144].

The MCA (Figure 4B) is one of the most studied cerebral vessels in fetal life [18,25,32,137,139,142]. Technical considerations for its evaluation are described by the International Society of Ultrasound in Obstetric and Gynecology Guidelines (ISUOG) 2021 [145]. It reflects the hemodynamic and metabolic status of the fetal brain in a wide variety of clinical and pathological scenarios, being the vessel of choice for studying the cerebroplacental ratio [26,145,146,147]. Besides its recognized value in the evaluation and management of fetal growth restriction and anemia [18,148], MCA has proved to be useful in other conditions, namely, in the study of CBF response to various exogenous substances, such as magnesium sulfate and anesthetics used during labor [149,150], and after laser intervention in twin-to-twin transfusion syndrome [151]. However, MCA has some limitations besides its recognized clinical interest. For example, its waveform alteration is an early manifestation of fetal hypoxia, but can be unreliable when fetal acidemia is eventually established [142]. Also, PI in MCA is reported to have a limited agreement between two observers, which leads to considerable variation in measurements in 30% of cases [145]. Some authors have attempted to study other cerebral vascular territories to improve the performance of Doppler studies in such clinical conditions [152,153,154]. J. Morales-Rosello et al. describe Doppler reference values of the fetal vertebral (Figure 4C) and middle cerebral arteries between 19 and 41 weeks of gestation [137]. Additionally, blood flow in the ICA (Figure 4D) has been explored; however, its clinical utility remains limited, and no reference curves of its Doppler indices have been published throughout pregnancy [140]. It is known that ICA Doppler indices, namely, impedance indices, might change in the same direction as MCA indices in the majority of physiological and some pathological conditions [6,155]. In this context, it is not evident if ICA is affected earlier in those circumstances. There is evidence showing that the brain-sparing effect and local autoregulation occur in a cranial–caudal and anterior–posterior direction [156]. However, some evidence against this has been recently published showing, for example, that autoregulation mechanisms are triggered in the vertebral artery before they are in the anterior cerebral circulation [137,156]. Given the complexity of fetal cerebral maturation and function towards the neonatal life, knowledge about blood flow in other vessels besides those extensively studied in the literature and clinical practice might be valuable and eliminate the risk of oversimplification.

Technically, an oblique section of the ICA can be observed on an axial view of the brainstem, anterior to the cerebral peduncles, on each side of the midline. Due to its proximity to the MCA, Woo et al. claim that it is not entirely possible to differentiate between the two arteries [157]. Although transabdominal US is recommended to evaluate CBF, one way to overcome this difficulty is to perform a transvaginal US, particularly if the presentation is engaged in the maternal pelvis [6].

Although Doppler evaluation of the ICA may follow the same guidelines as the evaluation of any vascular territory, specific technical issues have not been presented in the literature to date. There are no reference curves for this fetal vessel, and studies on its clinical value are inconclusive or have limited applicability.

## 4. Discussion

Due to the individual, social, and economic impact of irreversible brain damage that can occur during fetal development and in the transition to extrauterine life, through various expected and unexpected pathological conditions, improvement in knowledge about fetal brain autoregulation and blood flow redistribution is imperative. Although the majority of brain fetal vessels have already been explored, there is limited applicability of this knowledge for some of them. Apart from MCA, which is undoubtedly the artery more extensively studied, there are no reference curves for the majority of the other vascular territories, and the studies published on this subject are scarce, inconsistent, or lack strength. The construction of reference curves and percentiles for Doppler parameters in brain vessels, such as ICA, whose clinical interest has been described, would be available for comparative studies with several pathological conditions, validating its value for clinical practice.

## 5. Conclusions

Cerebral autoregulation consists of the intrinsic capacity of brain arterioles to contract and dilate in response to energy and oxygen deprivation, which is promptly modifiable by variations in blood pressure, chemical environment (pCO2 and pO2), metabolic environment (functional activation), and neurogenic activity [34]. As a vital organ, such a mechanism exists in the fetal and adult brain to reduce the risk of cerebral damage [33]. Myogenic vascular reactivity is attributed to the activity of vascular smooth muscle cell layers that possess motile properties [3]. The process of muscularization in brain arteries parallels the maturation of brain development, occurring in a centripetal direction and being only completed after term birth, which justifies why preterm newborns are at a higher risk of encephalopathy and cerebral hemorrhage compared with term newborns [3,4,99].

These adaptive mechanisms have an impact on fetal behavior and can be detected by fetal US, specifically through variations in the PI in cerebral arteries [21]. In hypoxemia, the net increase in CBF also triggers a redistribution of cerebral perfusion that favors the most metabolically demanding regions of the fetal brain at a particular stage of gestation [4]. The redistribution of blood flow also occurs at a systemic level, in which blood flow is centralized to favor vital organs, such as the brain, through an increase in peripheral vascular resistance and a parallel reduction in cerebral vascular resistance [66,67]. Despite these protective abilities, in situations of extreme oxygen deprivation, irreversible damage might occur with lifelong morbidity that is directly proportional to the duration and severity of oxygen deprivation and gestational age at the time of the insult [66,67]. For this reason, research on brain vascular characteristics is still justified.

Apart from an increase in blood flow achieved through autoregulation to maintain oxygen delivery, there are metabolic responses that mediate regional adaptation to oxygen deprivation. Hypoxia-homeostasis occurs in physiological as well as in pathological conditions, and in both prenatal and postnatal periods of brain development [75]. In acute oxygen deprivation, a well-established role exists for adenosine, PGE2, NO, cGMP, and specific neuropeptides, such as 5-HT, in the vasodilation of cerebral arterioles [64,75,83]. At the same time, fetal cortisol and catecholamine may be responsible for the shift towards an anaerobic state in the fetus [82,84]. In chronic hypoxia, VEGF and EPO induce multiple changes in cardiovascular and cerebrovascular regulation, ultimately leading to structural and functional modifications, with potential implications for fetal development [94]. At the intracellular level, signaling transduction mechanisms involved in cerebrovascular contractility and their changes with maturational development have been studied over the last few decades [98]. However, investigation on cerebrovascular maturation is complicated by the heterogeneity observed among vessels from different species, arteries of varying size, and among different vascular beds [98]. It is known that maturational changes in vascular smooth muscle cells involve (1) a transition in the Ca^2+^ source for contraction mechanisms from extracellular Ca^2+^ to an increasing dependence on SR as term approaches [98,105]; (2) a decrease in the quantity of MLCK available for phosphorylation of the MLC20, directly involved in contraction [127]; (3) an increase in the quantity of collagen in those vessels and (4) in actin cytoskeleton pathway gene expression towards term [129]. The net effect of all those molecular maturational changes will be the transition to a contractile phenotype in smooth muscle cells of cerebral fetal arteries, preparing the fetal brain for the transition to neonatal life [129]. The role of carotid arteries in this process has already been proven, related to their contractile ability, which defends distal cerebral vessels from injury associated with high blood flow [123,124]. It is expected that those changes be mirrored in spectral Doppler analysis of cerebral arteries.

The study of fetal CBF in vivo poses a challenge for four reasons: (1) it is an indirect observation, (2) the fetal brain is constantly changing its characteristics towards complete maturation, (3) maturation of the fetal brain will be concluded only after birth, and (4) it will change according to the maternal environment [139]. The MCA is one of the most studied cerebral vessels in fetal life [18,25,32,137,139,142]. Given the complexity of fetal cerebral maturation and function towards the neonatal life, knowledge about blood flow in other vessels besides those extensively studied in the literature and clinical practice might be valuable and eliminate the risk of oversimplification. There is some evidence in the literature about the potential utility of ICA in clinical practice. However, specific technical issues have not been presented in the literature until now; there are no reference curves for this fetal vessel, and studies on its clinical value are inconclusive or have limited applicability.

## Data Availability

The original contributions presented in this study are included in the article. Further inquiries can be directed to the corresponding author.

## Abbreviations

The following abbreviations are used in this manuscript:

5-HT	Serotonin
AA	Arachinoid acid
ANGII	Angiotensin II
ATP	Adenosine triphosphate
Ca^2+^	Calcium
cAMP	Cyclic adenosine monophosphate
CBF	Cerebral blood flow
CCs	Contractile cells
cGMP	Cyclic guanosine monophosphate
CO_2_	Carbon dioxide
COX	Cyclo-oxygenase
EEG	Electroencephalogram
EPO	Erythropoietin
EV	Ejection volume
FGF	Fibroblast growth factor
FLNA	Filamin isoform A
FLNB	Filamin isoform B
FBP1	Formin binding protein 1
HIF	Hypoxia-inducible factor
HIF-1α	Hypoxia-inducible factor 1-alpha
HIF-1β	Hypoxia-inducible factor 1-beta
HR	Heart rate
ICA	Internal carotid artery
ICAs	Internal carotid arteries
ISUOG	International Society of Ultrasound in Obstetric and Gynecology Guidelines
IP3	Inositol-1,4,5-trisphosphate
K^+^	Potassium
L	Length
MCA	Middle cerebral artery
MLC	Myosin light chain
MLCK	Myosin light chain kinase
mRNA	Messenger riboucleic acid
Na/K-ATPase	Sodium–potassium adenosine triphosphate desidrogenase
NE	Norepinephrine
NO	Nitric oxide
NOS	Nitric oxide synthase
NPY	Neuropeptide
pCO2	Carbone dioxide arterial partial pressure
PDGF	Platelet-derived growth factor
PI	Pulsatility index
pO2	Oxygen arterial partial pressure
PG	Prostaglandin
ROS	Reactive oxygen species
S	Section
SR	Sarcoplasmic reticulum
US	Ultrasound
V	Blood viscosity
VEGF	Vascular endothelial growth factor
